# Simultaneous quantification of triterpenoic acids by high performance liquid chromatography method in the extracts of gum resin of *Boswellia serrata* obtained by different extraction techniques

**DOI:** 10.1186/s13065-016-0194-8

**Published:** 2016-08-04

**Authors:** Neha Sharma, Vikram Bhardwaj, Samar Singh, Sheikh Abid Ali, D. K. Gupta, Satya Paul, Naresh K. Satti, Suresh Chandra, Mahendra K. Verma

**Affiliations:** 1 Analytical Chemistry Division (Instrumentation), CSIR-Indian Institute of Integrative Medicine, Canal Road, Jammu, 180001 Jammu and Kashmir India; 2 Natural Product Chemistry Division, CSIR-Indian Institute of Integrative Medicine, Jammu, 180001 India; 3 Bio-organic Chemistry Division, CSIR-Indian Institute of Integrative Medicine, Jammu, 180001 India; 4 Department of Biotechnology, CSIR-Indian Institute of Integrative Medicine, Jammu, 180001 India; 5 Department of Chemistry, University of Jammu, Jammu, 180006 India; 6 Genetic Resource and Agrotech, Division, CSIR-Indian Institute of Integrative Medicine, Jammu, 180001 India

**Keywords:** *Boswellia seratta*, HPLC, UV (DAD), Triterpenoic acids, ESI–MS (electrospray ionisation mass spectrometry)

## Abstract

**Background:**

*Boswellia serrata*, also known as Indian frankincense is a commercially important medicinal plant which has been used for hundreds of years as an Ayurvedic medicine for the attempted treatment of arthritis. It contains naturally occurring triterpenoic acids, called as boswellic acids (BA’s).

**Results:**

A highly reproducible High performance liquid chromatography-ultraviolet diode array detection (HPLC-UV-DAD) method was developed for the simultaneous determination and quantitative analysis of eight major triterpenoic acids in *Boswellia serrata* gum resin obtained by different extraction techniques. All the calibration curves exhibited good linear regression (R^2^ > 0.997) within the test ranges. The established method showed good precision and overall recoveries of the boswellic acids.

**Conclusions:**

The eight triterpenoic acids coded as BS-1 (11-keto-beta-boswellic acid), BS-2 (3-*O*-acetyl-11-keto-beta-boswellic acid), BS-3 (3-keto tirucallic acid), BS-4 (3-*O*-acetyl-alpha-tirucallic acid), BS-5 (3-*O*-acetyl-beta-tirucallic acid), BS-6 (alpha-boswellic acid), BS-7 (beta-boswellic acid) and BS-8 (3-*O*-acetyl-beta-boswellic acid) were isolated from the processed gum resin of *Boswellia serrata* by column chromatography. The proposed HPLC method is simple, reliable and has been very useful for the qualitative as well as quantitative analysis of boswellic acids in the gum resin of *Boswellia serrata*. The proposed method allows to quantify boswellic acids in appreciable amounts by HPLC-UV (DAD) method in the extracts and the available marketed formulations.

**Graphical abstract:**
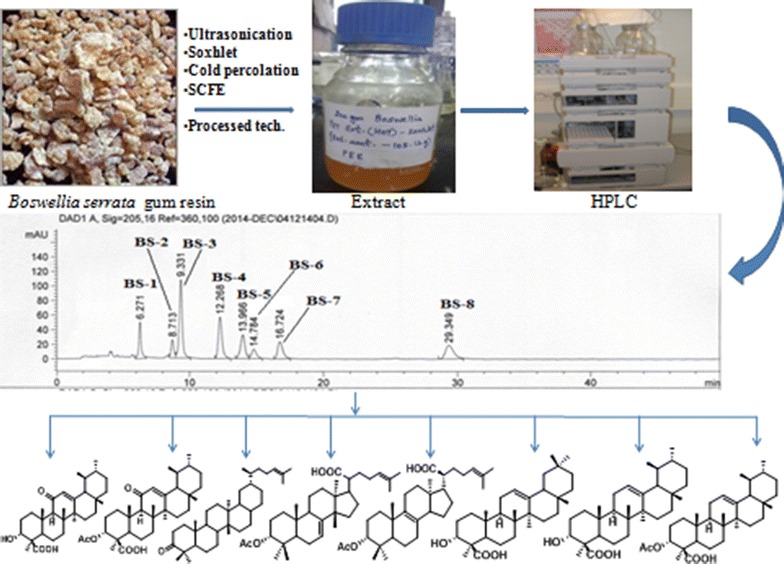
Isolation & separation of eight Triterpenoic acids from *Boswellia serrata*

## Background

Boswellin is methanolic/alcoholic extract of gum resin exudates of *Boswellia serrata*. Many preparations of the exudates of the plant are used in traditional medicines and have number of ethanobotanical applications.

**Fig. 1:**
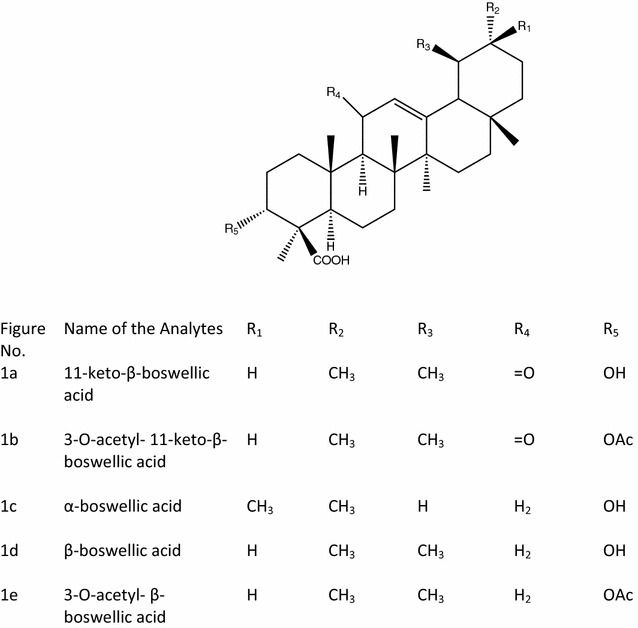
Chemical structure of  different Boswellic acids

**Fig. 2:**
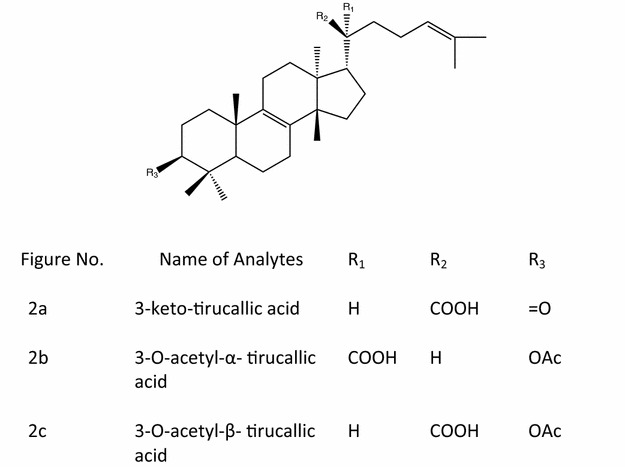
Chemical structure of different Tirucallic acids

**Fig. 3:**
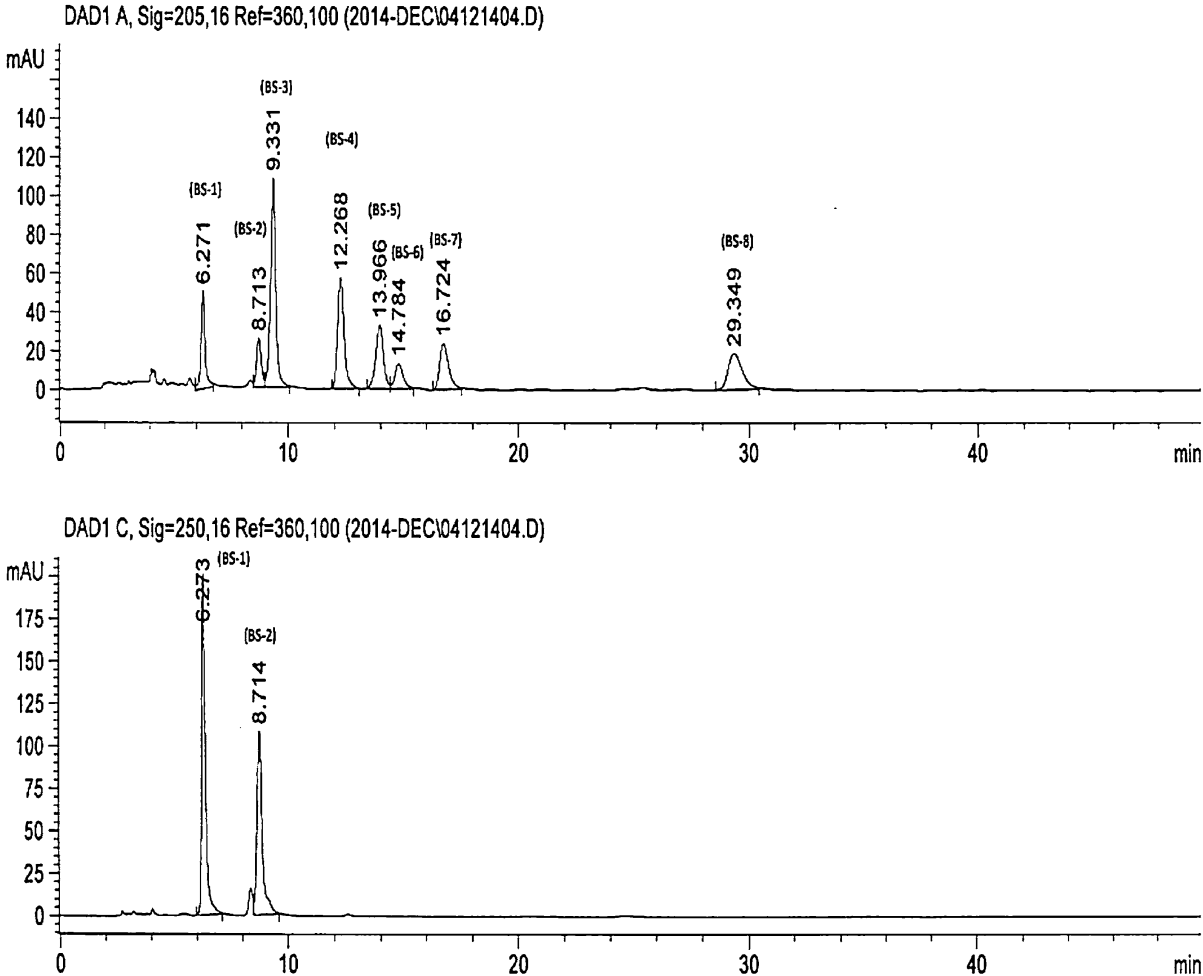
HPLC chromatogram showing the separation of different boswellic acids

*Boswellia serrata*, Linn F (Burseraceae) is commonly used in Indian system of medicine (Ayurveda) as anti-inflammatory, analgesic, anti-arthritic and anti-proliferative agent [1]. Clinical studies have revealed that *Boswellia serrata* plant may be effective in reducing diarrhoea in patients with inflammatory bowel disease [2]. A biopolymeric fraction of the plant has shown dose dependent immunostimulatory effect with respect to macrophage activation [3]. Anti-inflammatory activity of ethanolic extract of *Boswellia serrata* is primarily due to inhibition of leukotriene synthesis. In three small clinical trials boswellia was shown to improve symptoms of ulcerative colitis and Crohn’s disease. It has been reported that *Boswellia* plant is superior over mesalazine in terms of its alleged safety and benefit-risk ratio [4]. Both the gum resin and Acetyl Keto Boswellic acid (AKBA) exert moderate to low toxicity on the skin [5]. Eight major triterpenoic acids viz; 11-keto-beta-boswellic acid (Fig. 1a), 3-*O*-acetyl-11-keto-beta-boswellic acid (Fig. 1b), alpha-boswellic acid (Fig. 1c), beta-boswellic acid (Fig. 1d), 3-*O*-acetyl-beta-boswellic acid (Fig. 1e), 3-keto tirucallic acid (Fig. 2a), 3-*O*-acetyl-alpha-tirucallic acid (Fig. 2b) and 3-*O*-acetyl-beta-tirucallic acid (Fig. 2c) have been isolated from the gum resin of *Boswellia serrata*. These acids have been found to inhibit the synthesis of DNA, RNA and protein in human leukemia HL-60 cells in a dose dependent manner with IC_50_ values ranging from 0.6 to 7.1 µM. Among the eight above mentioned acids, 3-*O*-acetyl-11-keto-beta-boswellic acid have been reported to induce the most pronounced inhibitory effects on DNA, RNA and protein synthesis [6]. Experimental results suggest that boswellic acids are specific, non-redox inhibitors of leukotriene synthesis either by interacting directly with 5-lipoxygenase or by blocking its translocation [7–10]. *Boswellia* tree abundantly grows in dry hilly tracts of Gujarat and also in some parts of Madhya Pradesh states of India. This plant yields oleo-gum-resin which is used for variety of therapeutic purposes [1, 6, 11, 12, 13, 14, 15, 16, 17, 18, 19, 20]. Although the oleo-gum-resin which was a component of European Pharmacopoeia until the beginning of this century fell into oblivion with the use of synthetic drugs and still it is widely used in regions from North Africa to China. Alcoholic extract of Salai guggal (AESG) has been reported to possess anti-inflammatory and anti-arthritic activities in animals [13, 20] which were due to BA’s. Salai guggal contains 8–9 % essential oil, 20–23 % gum, and about 50 % resin [21, 22]. A non-phenolic fraction of *Boswellia serrata* has shown analgesic and psychopharmacological effects [23]. The studies have revealed the synergistic effect of boswellic acid mixture (BA) and glucosamine for anti-inflammatory and anti-arthritic activities in rats and this mixture has been found to give protection to gastric ulcer [24, 25]. A number of HPLC and HPLC–MS methods for analysis of triterpenoic acids are available in the literature [26–28] but there is no method so far available in the literature where simultaneous quantitative analysis of eight different triterpenoic acids (including Tirucallic acids) have been reported. We herein report a reliable, simple and an efficient method for the simultaneous determination and quantification of eight triterpenoic acids by HPLC-UV (DAD) method in the extracts obtained by different extraction techniques.

## Results and discussion

The HPLC method developed is a robust one and in order to develop the HPLC conditions for the separation of eight triterpenoic acids, isolated from the gum resin of *Boswellia serrata*, an artificial mixture of these triterpenoic acids was prepared. There were reasonable differences in the retention times (Table 1) of said boswellic acids which facilitated the quantification of these acids in the samples.

**Table 1:** Results of various chromatographic parameters for different Boswellic acids

Analyte	Retention time (min)	MDL (µg)	LOQ (µg)	Regression equation (Y = mx + b)	Correlation coefficient
11-keto-beta-boswellic acid (*BS-1*)	6.2	0.04	0.14	Y = 1.17752x + (−17.18153)	0.9999
3-*O*-acetyl-11-keto-beta-boswellic acid (*BS-2*)	8.6	0.13	0.44	Y = 9.38111e−1x +(−135.28362)	0.9999
3-*O*-keto-tirucallic acid (*BS-3*)	9.3	0.14	0.46	Y = 9.04242e−1x + (−97.40996)	0.9999
3-*O*-acetyl-alpha-tirucallic acid (*BS-4*)	12.2	0.26	0.86	Y = 1.01539x + (−73.37757)	0.9999
3-*O*-acetyl-beta-tirucallic acid (*BS-5*)	13.9	0.48	0.75	Y = 1.00921x + (−72.91381)	0.9998
Alpha- boswellic acid (*BS-6*)	14.7	0.93	1.43	Y = 6.05811e−1x + (−105.76374)	0.9983
Beta-boswellic acid (*BS-7*)	16.6	0.84	1.12	Y = 5.54846e−1x + (−126.78319)	0.9993
3-*O*-acetyl-beta-boswellic acid (*BS-8*)	29.2	0.96	1.26	Y = 4.65268e−1x + (−139.76004)	0.9998

Separation and detection of pentacyclic triterpenic acids, consisting of six boswellic acids, namely, 11-ketobeta-boswellic acid, 3-*O*-acetyl-11-keto- beta-boswellic acid, alpha-boswellic acid, beta-boswellic acid, 3-*O*-acetyl-alphaboswellic acid, and 3-*O*-acetyl-beta-boswellic acid was carried out by Subbaraju et al. whereas we were successful in the separation and detection of eight triterpenic acids including 3-keto-tirucallic acid, 3-*O*-acetyl-beta-tirucallic acid for the first time.

Quantification of these triterpenic acids were carried out for the first time in the extracts obtained from different extraction techniques. Gradient mobile phase was used by Subbaraju et al. whileas we used isocratic mobile phase [acetonitrile: 0.5 % acetic acid in water (95:5)] which is more reproducible and reliable. Proposed method exhibits calibration curves in the range of 2.0–120.0 μg (R2 > 0.998). This makes it more suitable for quality control of these acids in different commercial samples.

Shah et al. demonstrated the separation of only two acids; they have estimated that calibration curve was linear in the 11.66–58.30 and 6.50–32.50 μg/mL range for 11-KBA and A-11-KBA, respectively. The limits of detection were 2.33 and 1.30 μg/mL for 11-KBA and A-11-KBA, respectively. The mean recoveries were 98.24–104.17 % and 94.12–105.92 % for 11-KBA and A-11-KBA, respectively. The inter- and intra-day variation coefficients were less than 5 %. Where as in our proposed method, we have successfully separated eight different acids, calibration curve was observed linear in the range of 2.0–120 µg/mL, and the method detection limits were 0.04 and 0.13 μg/mL for 11-KBA and A-11-KBA, respectively. The overall recoveries were 98.6–100.2 % for 11-KBA and A-11-KBA. The assay precision ranged between 0.51 and 3.02 % whereas accuracy was between −2.37 and 6.44 % which shows the superiority of the method proposed.

Out of the eight triterpenoic acids used as standards in the study, boswellic acid and 3-*O*-acetyl-tirucallic acid showed separate peaks for α and β isomers in the HPLC–UV(DAD) chromatogram when separations were carried out on a non-chiral column (Merck, RP-18, 4 × 250 mm, 5 μm). Thus out of eight triterpenoic acids, two are actually mixtures of α and β isomers. The two peaks each for boswellic acid and 3-*O*-acetyl-tirucallic acid in the chromatograms were due to the α and β isomers. The responses of 11-keto boswellic acid, 3-*O*-acetyl-11-keto boswellic acid and 3-keto tirucallic acid on the same column were on the expected lines.

The results have revealed that 3-keto- tirucallic acid is a major constituent of the gum resin of *Boswellia serrata* which exhibited concentration of 26.4 % in the Processed Gum Resin (PGR). The results are the average of six samples taken up for quantification. Extracts obtained by different extraction techniques were subjected to quantification of eight major triterpenoic acids. The results have been detailed in Table 2.

**Table 2:** Results (%) of different boswellic acids in the extracts obtained by different extraction techniques

Compound	USE-E-RT	USE-E-HT	USE-PE-RT	USE-PE-HT	SOX-E	SOX-PE	CPE-1	CPE-2	SCFE	PGR
Extractive value (%)	25.28	54.55	19.30	34.14	47	49	27	22	3.9	57
BS-1	2.43	2.32	1.16	1.62	1.71	1.80	2.64	1.51	1.91	2.85
BS-2	1.79	1.24	1.33	1.79	0.91	2.23	2.21	2.01	4.51	5.64
BS-3	10.4	3.81	5.5	4.57	8.32	15.63	8.66	7.79	10.12	26.4
BS-4	1.08	0.62	1.6	1.77	0.93	2.1	1.32	1.91	2.73	2.72
BS-5	1.2	0.65	2.03	1.72	1.31	2.13	1.44	2.14	2.65	1.93
BS-6	7.07	4.57	7.24	4.81	2.88	9.86	6.05	8.77	6.01	8.58
BS-7	8.21	6.75	9.7	4.13	4.53	11.36	7.84	8.74	6.37	15.81
BS-8	3.3	2.36	5.3	7.57	4.15	9.34	4.51	8.02	7.87	9.18

*USE-E-RT* Ultrasonication in ethanol at room temperature, *USE-E-HT* Ultrasonication in ethanol at higher temperature, *USE-PE-RT* Ultrasonication in Petroleum ether at higher temperature, *USE-PE-HT* Ultrasonication in Petroleum ether at higher temperature, *SOX-E* Soxhlet extraction in ethanol, *SOX-PE* Soxhlet extraction in Petroleum ether, *CPE-1* Cold Percolation extraction in ethanol, *CPE-2* Cold Percolation extraction in Petroleum ether, *SCFE* Super critical fluid extraction, *PGR* Processed gum Resin

The compounds BS-1 and BS-2 showed maximum UV absorption at 250 nm while as other compounds BS-3 to BS-8 showed maximum UV absorption at 205 nm. Hence the quantification of the compounds was carried out at the aforesaid wavelengths (Fig. 3).

Proposed method is simple, precise, accurate and rapid as compared to the method previously reported [27]. In this method, eight boswellic acids were quantified in the different extracts obtained by different extraction techniques, which is not reported in the literature however several authors [29, 30] have reported the separation and detection of pentacyclic triterpenic acids, consisting of six boswellic acids, namely, 11-ketobeta-boswellic acid, 3-*O*-acetyl-11-keto-beta-boswellic acid, alpha-boswellic acid, beta-boswellic acid, 3-*O*-acetyl-alphaboswellic acid, and 3-*O*-acetyl-beta-boswellic acid whereas we were successful in the separation and detection of eight pentacyclic triterpenic acids including 3-keto-tirucallic acid, 3-*O*-acetyl-beta-tirucallic acid for the first time.

Different extraction techniques were employed for the extraction of eight boswellic acids by using different extraction solvents and conditions. It was observed that out of the different extraction techniques employed, the one in which the extract was treated with the base to yield acid rich fraction, a creamish powder called as processed gum resin (PGR) was an excellent technique for the isolation of majority of compounds with a total yield of 73.07 %. Soxhlet extraction with petroleum ether (SOX-PE) as extracting solvent came out to be the best technique next to processed gum resin with a total yield of 54.45 %. Total percent yield obtained from processed gum resin was 3.3 times more than that obtained from ultrasonication with ethanol at higher temperature (USE-E-HT), 1.7 times more than Supercritical fluid extraction (SCFE), 1.34 times than soxhlet extraction with petroleum ether (SOX-PE), 1.78 times than cold percolation in petroleum ether (CPE-2) and almost 2–3 times higher than what was obtained from other extraction techniques.

Since the plant and the isolated compounds are well known for their medicinal properties and uses in ayurveda, each of the extraction techniques can be employed for the targeted extraction of any particular compound from the plant extracts to get the maximum yield of the desired compound. Soxhlet extraction with petroleum ether has proved to be the best technique for the extraction and isolation of alpha boswellic acid (9.86 %). Similarly supercritical fluid extraction can be used for the isolation of acetyl-alpha tirucallic acid and acetyl-beta-tirucallic acid, processed gum resin can be employed in the case of acetyl-alpha-tirucallic acid and soxhelet extraction with petroleum ether is the best technique to be used for the isolation of acetyl-beta-boswellic acid.

## Experimental

### Instrumentation and reagents

All chemicals and reagents used in study were of high purity. HPLC grade acetonitrile and methanol were from Merck, India and HPLC grade water was from milli-Q water purification system. Analytical grade acetic acid from Merck India was used in the mobile phase whereas HPLC grade methanol was used for sample preparation. All the six triterpenoic acids, used as standards in the study, were isolated from the gum resin of *Boswellia serrata*. The purity of BA’s was established by HPLC whereas identity of these compounds was established by ESI–MS. The gum resin samples of *Boswellia serrata* were procured from local market of Ahmedabad city -Gujarat state of India and a specimen voucher of the samples was deposited in herbarium of the institute. Ultrasonication bath used was from Elma Sonic (Elma S 100 H) Singen, Germany.

### Isolation of markers from the gum resin

Ethanol (95 %) of 3 L was added in one kg of *B. serrata* gum resin and kept the same in a percolator up to the 24 h, similar process was repeated three times. All the solvent were filtered with the help of a filter paper, collected in 6 L conical flask and subjected to thin film rotavapor under reduced pressure at 40 °C to obtain a thick brown ethanolic extract with an extractive value of 49 %. NaOH (3 %) solution was mixed with the ethanolic extract to get a uniform emulsion. Filtration of aqueous portion was carried out with the help of a fine cloth and non-acidic part from the same was discarded by extracting it with the mixture of hexane/ethyl acetate (95:5). The total organic acids were precipitated out by acidifying it with 1 N HCl and were filtered. Distilled water was used to wash it sothat excess quantify of HCl can be removed from it. Aforesaid procedure was repeated to get precipitation of these acids and was dried at temperature below 50 °C in a vacuum oven. Finally it yielded 280 g of creamish powder i.e. the mixture of acids which contain triterpenoic boswellic acids. This mixture was subjected to column chromatography for the separation of individual acids. Hexane with increasing proportions of ethyl acetate was eluted through the column to collect different fraction. TLC was used to monitor these fractions; similar patterns showing fractions were pooled and evaporated to dryness over a rotavapor to get residues. These residues were subjected to crystallization and re-crystallization in appropriate organic solvents to yield pure crystals of different boswellic acids (BAs). Various spectroscopic techniques i.e. ^1^H NMR, ^13^C NMR and mass spectral data were employed to identify these compounds.

The quantification of eight BA’s was carried out in different extracts prepared by using different extraction techniques.

## Extraction methods

### Classical extraction by percolation

Cold percolation was carried out with two different solvents (ethyl alcohol and petroleum ether). 10 g of the plant material was taken and 40 mL of ethanol was added. The solvent was changed after 3 h, three repeated extractions were performed. These extractions were combined and processed for analysis. Similar procedure was adopted for extraction with petroleum ether. The extractive values obtained were 27 and 22 % in ethanol and petroleum ether respectively.

### Extraction under ultrasonic waves

For extraction under ultrasonic waves 10 g of plant material was taken, 40 mL of the solvent was added and the process was carried out with two different solvents (ethanol and petroleum ether) at two different temperatures (room temp. and at higher temp. of 45 °C). The extraction time was 2 h to achieve good extractive values. The marc left after filtration was extracted twice. All the three extracts were combined and dried under reduced pressure on a rotavapor. The extractive values for ultrasonication with ethanol at room temp and at higher temp were 25.28 and 54.55 % and for ultrasonication with petroleum ether at room temp and at higher temp were 19.3 and 34.14 % respectively. The extracts so obtained were analyzed for their contents by HPLC.

### Soxhlet’s extraction

Plant material (25 g) was extracted with two different solvents (ethyl alcohol and petroleum ether) of 200 mL on a water bath. After 5 h, the contents were filtered and the filtrate was evaporated to dryness under reduced pressure to yield semi dried extract. The extractive value obtained was 47 and 49 % for ethanol and petroleum ether.

### Supercritical fluid extraction (SFE)

SFE of plant material (1000 g) was carried out under the conditions wherein extraction pressure was 250 bars, temperature 45 °C and CO_2_ flow rate 20 kg/h, whereas pressure of Separator-1 was 100 bars and temperature was 40 °C and extraction was completed after 2 h. The extractive value was 3.9 %.

### Chromatographic conditions and apparatus

The HPLC system consisted of an Agilent series 1100 instrument equipped with a binary pump, an autosampler, an automatic electronic degasser, an automatic thermostatic column oven, a diode array detector, and a computer with Chemstation software (version 06.03 [509]) for data analysis. The HPLC separations were optimized using RP-18, Merck column (4 × 250 mm, 5 μm) where mobile phase consisted of an isocratic mixture of acetonitrile: 0.5 % acetic acid in water (95:5), The mobile phase was delivered at a flow rate of 0.8 mL/min. The column temperature was maintained at 30 °C to provide sharpness to the eluting peaks. The UV chromatograms were recorded at 205 and 250 nm. 11-keto-boswellic acid showed a peak at a retention time of 6.2 min in the HPLC-UV(DAD) chromatogram and similarly 3-*O*-acetyl-11-keto-boswellic acid, 3-keto-tirucallic acid, 3-*O*-acetyl-alpha-tirucallic acid, 3-*O*-acetyl-beta-tirucallic acid, alpha-boswellic acid, beta-boswellic acid and 3-*O*-acetyl-beta-boswellic acid exhibited peaks at retention times of 8.7, 9.3, 12.2, 13.9, 14.7, 16.7 and 29.3 min. respectively (Table 1).

### Preparation of standard solutions and sample solutions

#### Standard solutions

Eight BA’s i.e. BS-1, BS-2, BS-3, BS-4, BS-5, BS-6, BS-7 and BS-8 were properly weighed (5 mg) and each dissolved in 1 mL of HPLC grade methanol. All the eight standards were mixed in equal volumes and serially diluted. 10 µL of each of the above prepared mixtures was injected (n = 6) into the column for preparation of standard curves.

#### Sample solutions

Accurately weighed quantities of dried extract samples from gum resin of *Boswellia serrata* were dissolved in known volumes of HPLC grade methanol. The solutions were filtered through Millipore (0.45 µm) filters before injection into the HPLC system.

### MS analysis

MS studies on BA’s were carried out on Bruker ion trap (3000) Mass spectrometer because it is a straightforward and well suitable method for the ionization of polar and medium polar compounds. All the interface parameters were optimized and all the six standard solutions of BA’s were directly infused/injected into the mass spectrometer. The dry gas flow was 6 mL/min., nebulizer value 12 p.s.i and dry gas temperature was 320 °C. The mass range was 100–900 *m/z*, ICC target values were 8000 while the maximum accumulation time was set at 200 milli seconds. The six major BA’s exhibited molecular ion peaks at 471.2 ([M + H]^+^, BS-1), 513.3[M + H]^+^, BS-2) 453([M − H]^−^, BS-3), 497([(M − H]^−^, BS-4, BS-5), 455([M − H]^−^, BS-6, BS-7) and 496.9 ([M − H]^−^, BS-8) respectively.

### Quantification

Quantification of various gum resin samples of *Boswellia serrata* was carried out by external standard curve method. Quantification of eight BA’s 11-keto-beta- boswellic acid, 3-*O*-acetyl-11-keto-beta-boswellic acid, 3-keto tirucallic acid, 3-*O*-acetyl-alpha-tirucallic acid, 3-*O*-acetyl-beta-tirucallic acid, alpha-boswellic acid, beta-boswellic acid and 3-*O*-acetyl-beta-boswellic acid was carried out and excellent calibration curves were obtained for all the boswellic acids in the concentration range of 2.0–120.0 µg (R^2^ > 0.998). The recovery studies were within the concentration range of calibration curves.

### Validation of the LC method

#### Linearity

Linearity of the detector response was determined on the basis of calibration curves. In the present study, linearity of eight major triterpenoic acids (BA’s) was studied in the concentration range of 2.0–120 µg by using standard solutions of different boswellic acids respectively. Regression analysis co-efficient (*r*) was greater than 0.998.

#### Relative recovery

The relative recovery of the method was estimated by spiking 0.5 mg each of the eight boswellic acids in 500 mg of the extract. The recovery studies were carried out at three different concentrations 10, 50, 90 µg/mL which revealed that the recoveries of boswellic acids were in the range of 97.8–100.2 % (Table 3).

**Table 3:** Results of relative recovery analysis for different boswellic acids

Analyte	Actual concentration (μg/mL)	Detected concentration (μg/mL)	% RSD	Recovery %
11-Keto-beta-boswellic acid	10.0	9.86 ± 0.21	2.14	98.6
	50.0	49.70 ± 0.51	1.02	99.4
	90.00	90.25 ± 0.82	0.9	100.2
3-*O*-acetyl-11-keto-beta-boswellic acid	10.0	9.90 ± 0.20	2.02	99.0
	50.0	50.06 ± 0.72	1.43	100.1
	90.00	89.96 ± 0.70	0.77	99.9
3-*O*-keto-tirucallic acid	10.0	9.91 ± 0.23	2.32	99.1
	50.0	49.82 ± 0.44	0.88	99.6
	90.00	89.92 ± 0.72	0.80	99.9
3-*O*-acetyl-alpha-tirucallic acid	10.0	9.78 ± 0.15	1.54	97.8
	50.0	49.80 ± 0.73	1.46	99.6
	90.00	89.75 ± 0.50	0.55	99.7
3-*O*-acetyl-beta-tirucallic acid	10.0	9.91 ± 0.21	2.19	99.1
	50.0	49.76 ± 1.19	2.41	99.52
	90.00	88.92 ± 0.93	1.05	98.8
Alpha-boswellic acid	10.0	9.91 ± 0.25	2.62	99.1
	50.0	49.68 ± 1.25	2.51	99.36
	90.00	89.48 ± 0.57	0.63	99.42
Boswellic acid	10.0	9.98 ± 0.13	1.31	99.8
	50.0	49.77 ± 0.84	1.68	99.5
	90.00	89.88 ± 0.82	0.91	99.8
3-*O*-acetyl-beta- boswellic acid	10.0	9.93 ± 0.16	1.61	99.3
	50.0	49.51 ± 0.10	0.20	99.0
	90.00	89.96 ± 0.64	0.71	99.9

#### Precision and accuracy

Intra-day precision and accuracy was determined by assaying standard solutions of eight boswellic acids at three different concentrations which fall within the range of the calibration curve. The overall intraday and interday precisions (% RSD) were set as less than 10 % whereas accuracy (% RE) was less than ±10 %. The values for interday precision and accuracy for all the eight major boswellic acids were measured by analysis of the standard solutions at three concentrations on three different days. The calculated RSD and RE values from repeated measurements are summarized in Table 4. The assay precision ranged between 0.51 and 3.02 % whereas accuracy was between −2.37 and 6.44 %.

**Table 4:** Results of interday and intraday precision (RSD %) and accuracy (RE %) for different boswellic acids

Analyte	Actual concentration (μg/ml)	Detected concentration (mean ± SD, n = 6)		RSD (%)		RE (%)	
		Interday	Intraday	Interday	Intraday	Interday	Intraday
11-Keto-beta-boswellic acid	8.0	7.90 ± 0.10	8.04 ± 0.15	1.26	1.86	−1.25	0.50
	25.0	24.90 ± 0.60	26.43 ± 0.66	2.40	2.49	−1.25	5.72
	100.00	99.60 ± 2.88	100.35 ± 2.84	2.89	2.83	−0.40	0.35
3-*O*-acetyl-11-keto-beta-boswellic acid	8.0	7.96 ± 0.20	8.21 ± 0.20	2.51	2.43	−0.50	2.62
	25.0	25.08 ± 0.32	26.50 ± 0.74	1.27	2.79	0.32	6.00
	100.00	99.70 ± 2.84	100.61 ± 2.97	2.84	2.95	−0.30	0.61
3-*O*-keto-tirucallic acid	8.0	8.09 ± 0.10	8.02 ± 0.24	1.23	2.99	1.12	0.25
	25.0	25.05 ± 0.66	26.61 ± 0.80	2.63	3.00	0.20	6.44
	100.00	97.90 ± 2.96	100.94 ± 2.94	3.02	2.91	−2.1	0.94
3-*O*-acetyl-alpha-tirucallic acid	8.0	8.03 ± 0.10	7.96 ± 0.11	1.24	1.38	0.37	−0.5
	25.0	25.06 ± 0.13	24.89 ± 0.24	0.51	0.96	0.24	−0.44
	100.00	100.21 ± 0.70	99.83 ± 0.76	0.69	0.76	0.21	−0.17
3-*O*-acetyl-beta-tirucallic acid	8.0	7.98 ± 0.16	7.92 ± 0.16	2.0	2.02	−0.25	−1.00
	25.0	26.29 ± 0.64	25.43 ± 0.61	2.43	2.39	5.16	1.72
	100.00	99.52 ± 2.80	100.63 ± 2.52	2.81	2.50	−0.48	0.63
Alpha-boswellic acid	8.0	7.85 ± 0.07	7.82 ± 0.11	0.89	1.40	−1.87	−2.25
	25.0	24.73 ± 0.22	25.61 ± 0.66	0.88	2.57	−1.08	2.44
	100.00	98.89 ± 0.90	100.41 ± 1.67	0.91	1.66	−1.11	0.41
Beta-boswellic acid	8.0	7.94 ± 0.10	7.96 ± 0.22	1.25	2.76	−0.75	−0.5
	25.0	24.88 ± 0.72	25.31 ± 0.40	2.89	1.58	−0.48	1.24
	100.00	100.13 ± 1.93	97.63 ± 2.88	1.92	2.94	0.30	−2.37
3-*O*-acetyl-beta-boswellic acid	8.0	8.03 ± 0.18	8.09 ± 0.24	2.24	2.96	0.37	1.12
	25.0	25.81 ± 0.60	25.68 ± 0.62	2.32	2.41	3.24	2.72
	100.00	100.18 ± 2.53	99.37 ± 2.69	2.52	2.70	0.18	−0.63

### Limit of detection and limit of quantification

Method detection limit (MDL) was established as the amount of analyte that provides a signal-to-noise ratio of 3. The MDLs were 0.04, 0.13, 0.14, 0.26, 0.48, 0.93, 0.84 and 0.96 µg for 11-keto-beta-boswellic acid, 3-*O*-acetyl-11-keto-beta-boswellic acid, 3-keto-tirucallic acid, 3-*O*-acetyl-alpha-tirucallic acid, 3-*O*-acetyl-beta-tirucallic acid, alpha-boswellic acid, beta-boswellic acid and 3-*O*-acetyl-beta-boswellic acid respectively. Limits of quantification (LOQ) were established as the amount of analyte that provides a signal-to-noise ratio of 10. The limit of quantification (LOQ) was 0.14, 0.44, 0.46, 0.86, 0.75, 1.43, 1.12 and 1.26 µg for for 11-keto-beta-boswellic acid, 3-*O*-acetyl-11-keto-beta-boswellic acid, 3-keto-tirucallic acid, 3-*O*-acetyl-alpha-tirucallic acid, 3-*O*-acetyl-beta-tirucallic acid, alpha-boswellic acid, beta-boswellic acid and 3-*O*-acetyl-beta-boswellic acid respectively.

## Conclusions

The proposed HPLC method is simple, reliable and has been very useful for the qualitative as well as quantitative analysis of boswellic acids in the gum resin of *Boswellia serrata*. The method allows to quantify boswellic acids in appreciable amounts by HPLC-UV (DAD) method. In case of those boswellic acids which exhibited isomeric peaks, both the isomeric peaks were considered for all measurements. This method has allowed us to fix the acceptance and rejection criteria for the selection of gum resin samples of the plant which is being used by pharmacologists to study its various attributes.

## References

[1] Pandey RS, Singh BK, Tripathi YB (2005). Extract of gum resin of *Boswellia serrata* inhibits lipopolysaccharide induced nitric oxide production in rat macrophages along with hypolipidemic property. Indian J Exp Biol.

[2] Borrelli F, Capasso F, Capasso R, Ascione V, Aviello G, Rocco L, Izzo A (2006). Effect of *Boswellia serrata* on intestinal motility in rodents: inhibition of diarrhoea without constipation. Br J Pharmacol.

[3] Khajuria A, Gupta A, Suden P, Singh S, Malik F, Singh J, Gupta BD, Suri KA, Srinivas VK, Ella K, Qazi GN (2008). Immunomodulatory activity of biopolymeric fraction BOS 2000 from *Boswellia serrate*. Phytother Res.

[4] Kiela PR, Midura AJ, Kuscuoglu N, Jolad SD, Sólyom AM, Besselsen DG, Timmermann BN, Ghishan FK (2005). Effects of Boswellia serrata in mouse models of chemically induced colitis. Am J Physiol Gastrointest Liver Physio.

[5] Hozumi H, Asanuma M, Miyazaki I, Fukuoka S, Kikkawa Y, Kimoto N, Kitamura Y, Sendo T, Kita T, Gomita Y (2008). Comparison of the irritation potentials of Boswellia serrata gum resin and of acetyl-11-keto-β-boswellic acid by in vitro cytotoxicity tests on human skin-derived cell lines. Toxicol Lett.

[6] Shao Y, Chin H, Badmaev CT, Ma CK, Huang V (1998). W MT: Inhibitory activity of Boswellic acids from *Bosweliia serrata* against human leukemia HL-60 cells in culture. Planta Med.

[7] Ammon HP, Mack T, Singh GB, Safayhi H (1991). Inhibition of leukotriene B4 formation in rat peritoneal neutrophils by an ethanolic extract of gum resin exudate of *Boswellia serrate*. Planta Med.

[8] Safayhi H, Sailer ER, Ammon HP (1995). Mechanism of 5-lipoxygenase inhibition by acetyl-11-keto-b-boswellic acid. Mol Pharmacol.

[9] Sailer ER, Subramanian LR, Rall B, Hoernlein RF, Ammon HP, Safayhi H (1996). Acetyl 11-keto-b-boswellic acid (AKBA): Structure requirements for binding and 5-lipoxygenase inhibitory activity. Br J Pharmacol.

[10] Bhargava GG, Negi JJ, Ghua HRD (1978). Studies on the chemical composition of salai gum. Indian For.

[11] Sharma A, Mann AS, Gajbhiye V, Kharya MD (2007). Phytochemical profile of *Boswellia serrata*: an overview. Pharmacogn Rev.

[12] Marinetz D, Lohs K, Janzen J (1988) Weihrauch and myrrhe*. Bedeutung Botanik Chemir Wiss Vertges Stuttgast*, p 153

[13] Singh GB, Atal CK (1986). Pharmacology of an extract of Salai guggal ex-*Boswellia serrata*, a new non steroidal anti-inflammatory agent. Agents Action.

[14] Sharma ML, Bani S, Singh GB (1989). Anti-arthritic activity of boswellic acids in bovine serum albumin (BSA)-induced arthritis. Int J Immunopharmacol.

[15] Gupta I, Gupta V, Parihar A, Gupta S, Ludtke R, Safayhi H, Ammon HP (1998). Effects of *Boswellia serrata* gum resin in patients with bronchial asthma: results of a double-blind, placebo controlled, 6-week clinical study. Eur J Med Res.

[16] Chopra RN, Nayar SL, Chopra IC (1956). Glossary of Indian medicinal plants.

[17] Gupta I, Parihar A, Malhotra P, Gupta S, Ludtke A, Safayhi H, Ammon HP (2001). Effects of gum resin of Boswellia serrata in patients with chronic colitis. Planta Med.

[18] Gerhardt H, Seifert F, Buvari P, Vogelsang H, Repges R (2001). Therapy of ctive Crohn disease with *Boswellia serrata* extract H-15. Z Gastroenterol.

[19] Reddy GK, Dhar SC, Singh GB (1987). Urinary excretion of connective tissue metabolites under the influence of a new non-steroidal anti-inflammatory agent in adjuvant induced arthritis. Agents Actions.

[20] Safayhi H, Mack T, Sabieraj J, Anazodo MI, Subramanian LR, Ammon HP (1992). Boswellic acids: novel, specific, nonredox inhibitors of 5-lipoxygenase. J Pharmacol Exp Ther.

[21] Pardhy RS, Bhattacharya SC (1978). Boswellic acid, acetyl-boswellic acid and 11-keto-boswellic acid, four pentacyclic triterpenic acids from the resin of *Boswellia serrata* Roxb. Indian J Chem.

[22] Kumar A, Saxena VK (1979). TLC and GLC studies on the essential oil from *Boswellia serrata* leaves. Indian Drugs.

[23] Menon MK, Kar A (1971). Analgesic and psychopharmacological effects of the gum resin of *Boswellia serrate*. Planta Med.

[24] Singh S, Khajuria A, Taneja SC, Khajuria RK, Singh J, Qazi GN (2007). Boswellic acids and glucosamine show synergistic effect in preclinical anti-inflammatory study in rats. Bioorg Med Chem Lett.

[25] Singh S, Khajuria A, Taneja SC, Khajuria RK, Singh J, Johri RK, Qazi GN (2008). The gastric ulcer protective effect of boswellic acids, a leukotriene inhibitor from *Boswellia serratta* in rats. Phytomedicine.

[26] Reising K, Meins J, Bastian B, Eckert G, Mueller WE, Schubert MZ, Tawab MA (2005). Derermination of boswellic acids in brain and plasma by high performance liquid chromatography tandem mass spectrometry. Anal Chem.

[27] Shah AS, Rathod SI, Suhagia NB, Pandya SS, Parmar VK (2008). A simple high-performance liquid chromatographic method for the estimation of boswellic acids from the market formulations containing *Boswellia serrata* extract. J Chromatogr Sci.

[28] Frank A, Unger M (2006). Analysis of frankincense from various *Boswellia* species with inhibitory activity on human drug metabolising cytochrome P450 enzymes using liquid chromatography mass spectrometry after automated on-line extraction. J Chromatogr A.

[29] Subbaraju GV, Sridhar P, Ramakrishna S, Sreemannarayana A, Vanisree M, Babu SK (2004). Isolation and HPLC estimation of six boswellic acids from Boswellia serrata extract. Asian J Chem.

[30] Kathleen G, Jan H, Gert FOW, Manfred SZ, Mona AT (2013). In vitro metabolism, permeation, and brain availability of six major boswellic acids from Boswellia serrata gum resins. Fitoterapia.

